# The similarity of inherited diseases (I): clinical similarity within the phenotypic series

**DOI:** 10.1186/s12920-021-00900-7

**Published:** 2021-02-23

**Authors:** Alessio Gamba, Mario Salmona, Gianfranco Bazzoni

**Affiliations:** Department of Biochemistry and Molecular Pharmacology, Istituto Di Ricerche Farmacologiche Mario Negri IRCCS, Via Mario Negri 2, 20156 Milano, Italy

**Keywords:** Gene mutations, Inherited diseases, Disease phenotypes, Differential diagnosis, Network analysis, Graph theory

## Abstract

**Background:**

Mutations of different genes often result in clinically similar diseases. Among the datasets of similar diseases, we analyzed the ‘phenotypic series’ from Online Mendelian Inheritance in Man and examined the similarity of the diseases that belong to the same phenotypic series, because we hypothesize that clinical similarity may unveil shared pathogenic mechanisms.

**Methods:**

Specifically, for each pair of diseases, we quantified their similarity, based on both number and information content of the shared clinical phenotypes. Then, we assembled the disease similarity network, in which nodes represent diseases and edges represent clinical similarities.

**Results:**

On average, diseases have high similarity with other diseases of their own phenotypic series, even though about one third of diseases have their maximal similarity with a disease of another series. Consequently, the network is assortative (i.e., diseases belonging to the same series link preferentially to each other), but the series differ in the way they distribute within the network. Specifically, heterophobic series, which minimize links to other series, form islands at the periphery of the network, whereas heterophilic series, which are highly inter-connected with other series, occupy the center of the network.

**Conclusions:**

The finding that the phenotypic series display not only internal similarity (assortativity) but also varying degrees of external similarity (ranging from heterophobicity to heterophilicity) calls for investigation of biological mechanisms that might be shared among different series. The correlation between the clinical and biological similarities of the phenotypic series is analyzed in Part II of this study^1^.

## Background

After the early reports of gene mutations as causes of inherited diseases (D), the ‘one gene-one disease’ paradigm became widely accepted. According to the paradigm, a mutation in one gene results in the production of a dysfunctional disease gene product (DGP), in general a protein, which in turn (in a direct or indirect way) causes the disease phenotypes (DP) that characterize the D [1]. Yet, with time, it has become clear that the paradigm has exceptions. For instance, in locus heterogeneity, mutations in different (and often seemingly unrelated) genes cause similar (if not identical) D [2]. These exceptions raise important questions. In particular, how can we explain (in mechanistic terms) the occurrence of genetically different—but clinically—similar D? One possibility is that the different molecular activities, which are usually carried out by normal proteins (i.e., the non-mutated counterparts of the DGP), converge towards a shared biological response [3], often as members of the same protein complex [4]. Thus, the mutations of different genes, which cause clinically similar D, could be thought of as alternative biochemical means of altering the same biological function (and/or protein complex) and eventually producing the same clinical phenotype. However, before analyzing all the individual types of clinically similar D, we need an objective and measurable means of defining the general concept of clinical similarity.

Nowadays, with ever-growing lists of D, we need at least two major tools to study D similarity. First, we need broad databases, which report all the known D, as well as detailed databases, which report all the clinical manifestations (the DP) of each D. Second, we need efficient algorithms to quantify the degree of similarity among D. In general, the identification of clinically similar D is difficult, because of the way D are defined as ‘similar’. In particular, classifications of similar D that rely on clinical judgment are subjective by definition, no matter how authoritative the source of judgment is. Nonetheless, defining similarity has become somehow easier, thanks to the recent availability of databases of D and DP. In addition, efficient similarity-computing algorithms have been implemented and validated [5]. One of the catalogues of clinically similar (but molecularly different) D are the Phenotypic Series (PS) of Online Mendelian Inheritance in Man (OMIM). With its coverage of more than 3,000 D (grouped into about 400 PS), the OMIM list of PS is likely one of the most complete, authoritative and reliable sources of similar D [6]. In addition, another database, namely Human Phenotype Ontology (HPO), annotates each OMIM-derived D with a detailed list of DP, according to a controlled and hierarchical vocabulary [7]. Thus, we asked whether it would be possible to use the available databases and algorithms to quantify the degree of clinical similarity among presumptively similar D, based on the objective criteria of which DP these D share. We found that the vast majority of D has indeed high similarity with the other D in their own PS. However, we also found that numerous D (in spite of a high intra-PS similarity) are highly similar to D of different PS as well, thus unveiling an unexpectedly complex picture of D similarity.

**Fig. 1:**
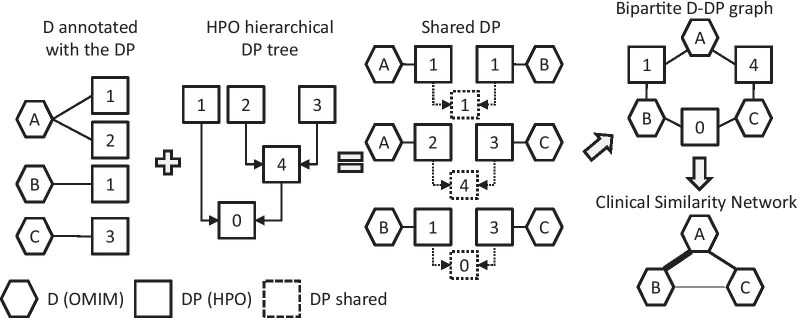
Assembly of a clinical similarity network. The D from OMIM (*hexagons*) are annotated with the DP from HPO (*squares*). As shown in the simplified hierarchical tree, the DP term 4 is the common ancestor of terms 2 and 3, whereas 0 is the root. Then, a typical similarity-searching algorithm retrieves all the possible D_i_ − D_j_ pairs and the DP annotations that the two D share (*dotted squares*). The shared annotations can be either the identical DP that annotates both D or another term (often, the most informative common ancestor of two different DP). For instance, 1 is shared (by A and B) as identical term, while 4 is shared (by A and C) as common ancestor of 2 and 3. With these criteria, even two dissimilar D (e.g., B and C) will eventually share at least some (poorly informative) term (in this example, the root 0). At the end of the search, a non-weighted D-DP bipartite graph is assembled. Then, from the bipartite graph, a clinical similarity network is derived, by linking all the D that share DP annotations (the *thickness* of the edge being proportional to the similarity coefficients of the two linked D). Note that, for simplicity’s sake, the figure does not indicate the PS and that each pair of D is shown to share only one DP annotation

Furthermore, the high number of D and DP requires appropriate analytical methods, if one desires to scale up the study of similarity from a local level (few pairs of D at a time) to a more global level (the whole ensemble of all the known D). One of these methods is network analysis. Networks are used in several disciplines, including medicine and biology [8]. We surmised that the vast complexity of all the D similarities could be suitably analyzed by means of a bipartite D-DP graph, which displays the links between D and DP. Then, from the D-DP bipartite graph, one can derive a network of clinical similarity (Fig. 1). In general, in a similarity network, any two nodes are linked by a weighted edge, which indicates that they are similar (the weight being proportional to the degree of similarity). Thus, if the D that belong to the same PS are clinically similar to each other, we expected them to be linked in a network of clinical similarity as well. The statement is not a tautology, because the assembly of such a similarity network does not rely on the clinical judgment that was used to assemble the PS, but rather relies on the objective sharing of DP among D, as derived from an external and independent source (e.g., HPO). Indeed, we found that the D that belong to the same PS are heavily inter-connected in the Disease-Disease Similarity Network-Clinical (DDSN-C), which has been assembled with minor modifications of the general strategy outlined in Fig. 1 (as detailed in the Methods).

If the nodes of a network (here, the D), which possess a given property (here, the PS to which the D belong), tend to link to each other, then the network is *assortative* with respect to that property. In other words, we found that the DDSN-C is assortative with respect to the PS, thus further supporting the general conclusion that the PS are homogeneous clusters of clinically similar D. Nevertheless, we also found that such PS-based linkages influence in different ways the structure of the network. Specifically, we observed that the individual PS (besides being assortative) vary in their relations with the other PS, ranging from *heterophobic* PS—whose D have low similarity (and thus few links) with D of other PS—to *heterophilic* PS whose D have high similarity (and thus several links) with D of other PS. Thus, this study provides strong quantitative evidence that the PS (in parallel to their intra-PS homogeneity) also display variable degrees of inter-PS similarity.

## Methods

### The D and PS from OMIM

The morbid map was downloaded from the OMIM website and the full list of the PS was kindly provided by the OMIM team [6]. Only D with known molecular basis were retained for analysis. In contrast, we did not examine D with unknown gene defect, D with unknown mutation and D caused by multiple genes. We also excluded the D defined as susceptibility to multi-factorial disorders, non-D and unconfirmed D.

### The DP from HPO

The DP associated with the OMIM-encoded D were retrieved from the HPO website [7]. The HPO terms are arranged hierarchically, starting from the common ancestor HP:0000001 (All) and its five ‘children’ terms, i.e., HP:0000118 (Phenotypic abnormality), HP:0000005 (Mode of inheritance), HP:0012823 (Clinical modifier), HP:0040006 (Mortality/Aging) and HP:0040279 (Frequency). In this study, only the 13,427 descendants of HP:0000118 (and not the descendants of the other four children of All) have been used, because they more selectively identify the DP. Finally, an IC (normalized in the 0–1 range), which is inversely proportional to the number of D that the DP annotates in HPO, was attributed to each DP.

### Clinical annotations of the D from DO

The OMIM-encoded D were classified according to the following major subdivisions of the Disease Ontology (DO) hierarchical tree [9]. (i) The eight direct descendants of the common ancestor DO:0000004 (Disease), i.e., Syndrome, Genetic disease, Physical disorder, Disease by infectious agent, Disease of metabolism, Disease of mental health, Disease of cellular proliferation and Disease of anatomical entity. (ii) The twelve direct descendants of DO:0000007 (Disease of anatomical entity), i.e., Diseases of the Cardiovascular, Endocrine, Gastrointestinal, Hematopoietic, Immune, Integumentary, Musculoskeletal, Nervous, Reproductive, Respiratory, Thoracic and Urinary System.

### The similarity score

After assembling all the possible D_i_ − D_j_ pairs, the *M* DP that annotate D_i_ and the *N* DP that annotate D_j_ were retrieved. Then, for each D_i_ − D_j_ pair, each of the resulting *M* * *N* DP pair (i.e., the *m*th DP annotating D_i_ and the *n*th DP annotating D_j_) were compared to identify the shared DP, which is either the highest specificity ancestor of the two DP (if different) or the DP itself (if identical). Finally, for each D_i_ − D_j_ pair, the IC of all the shared DP were averaged, to output a score of clinical similarity between D_i_ and D_j_.

### The D-DP bipartite graph

The bipartite D-DP graph is composed of D and DP nodes, as well as D-DP edges. To assemble the D-DP graph, each D could be simply linked with all its DP annotations in the HPO dataset. However, the following should be kept into account. First, the D-DP graph is prerequisite for assembling the DDSN-C (because any two D become linked mutually in the latter, if they are linked to the same DP in the former). Second, the DP annotations of the D can be at different distances from the root (in the hierarchical tree of HPO). Thus, to assemble the D-DP, in addition to the HPO-provided DP annotations, we also included DP (still from the HPO dictionary) that might identify occurrences of high clinical similarity between two D that are not annotated with an identical DP. Specifically, for any D_i_ − D_j_ pair, we identified—as shared DP_(i,j)_—not only an identical DP (i.e., a DP that annotates—according to HPO—both D_i_ and D_j_) but also a highly similar DP, according to two additional criteria. First, DP_(i,j)_ is a first-degree ancestor of the two different DP, which annotate D_i_ and D_j_ in HPO. Second, DP_(i,j)_ is the DP that annotates D_i_ according to HPO and is also the first-degree ancestor of the DP that in HPO annotates D_j_ (or the other way around).

### The DDSN-C

Thus, the DDSN-C was derived from the bipartite D-DP graph by linking, with an edge, a given pair of D nodes, anytime the two D share one DP_(i,j)_ as defined above. Because of this initial step, any two D, which share more than one DP, are linked by more than one edge. Thus, to obtain a more compact version of the DDSN-C, the multiple edges (connecting a given pair of D) were merged into just one edge. Finally, each resulting edge was assigned a weight *w*, which is the average IC of all the DP that link the two D.

### Assortativity analysis of the DDSN-C

On the assumption that each node in the DDSN-C is characterized by the PS of the corresponding D, the correlation of such node characteristics with the structure of the DDSN-C was quantified by calculating the dyadicity D and heterophilicity H of each PS, as described [10]. Briefly, each PS was regarded as a binary characteristic, i.e., a property that a given node in the network either does or does not possess. Then, all the dyads were examined (each dyad being composed of an edge plus its two end nodes). Specifically, with respect to each property (i.e., a given PS_x_), the observed number of homogeneous (PS_x_/PS_x_) and heterogeneous (PS_x_/PS_non-x_) dyads was calculated. In parallel, the number of dyads was calculated, which could be expected, if the property were distributed randomly in the DDSN-C. The observed-to-expected ratios of homogeneous and heterogeneous dyads are the values of D and H for that PS, respectively. If D > 1, the property is dyadic (or antidyadic, if D < 1) and thus homogeneous nodes connect mutually more frequently (less frequently, if antidyadic) than expected for a random distribution. Furthermore, if H > 1, the property is heterophilic (heterophobic, if H < 1) and thus heterogeneous nodes connect to each other more frequently (less frequently, if heterophobic) than expected for a random distribution.

## Results

### General aim, methodological approach and expected findings

We undertook this study to evaluate whether inherited D, which have been defined similar based on subjective and qualitative judgment (and thus included in the same PS), can be defined similar based on objective and quantitative criteria as well. To this purpose, we first calculated a score of binary similarity for each pair of D, based on both number and IC of the shared DP. Then, we assembled a bipartite D-DP graph and derived a network of clinically similar D (the DDSN-C). Finally, we evaluated the distribution of the PS within the DDSN-C by assortativity and dyad analysis.

We expected that pairs of D, which belong to the same PS, have high similarity scores (compared with randomly paired D) and are linked in the DDSN-C, such that the PS emerge as dyadic properties (dyadicity referring to pairs of nodes that are linked and have the same property). Conversely, we expected that pairs of D, which belong to different PS, have low similarity scores and are not linked in the DDSN-C. As the D pairs (linked and non-linked alike) that belong to different PS comprise the majority of all the theoretically possible node pairs in the DDSN-C, we also expected that the PS emerge not only as dyadic but also as heterophobic properties (heterophobicity referring to pairs of nodes that avoid links and do not have the same property).

### The OMIM database identifies molecularly characterized D

We first searched all the human D with known molecular basis. To this purpose, we retrieved the morbid map of OMIM, which reports both D and DGP. We focused on a subset of 4,193 molecularly characterized D and 3,211 DGP. The discrepancy between D and DGP count is mostly due to allele and locus heterogeneity. In allele heterogeneity, the same DGP causes more D. For instance, the DGP encoded by the *COL2A1* gene (a collagen subunit) causes 14 collagenopathies, including the *Stickler syndrome*. Conversely, in locus heterogeneity, more DGP cause the same D. For example, 21 DGP cause *Colorectal cancer, somatic*. Thus, with thousands of D, the morbid map provided a sufficiently wide dataset to start a global analysis of D similarity. In the subsequent sections, however, we considered neither type of heterogeneity. Rather, we focus on the PS, in which different DGP cause similar (not necessarily identical) D.

### The OMIM database identifies the PS as groups of clinically similar D

**Fig. 2:**
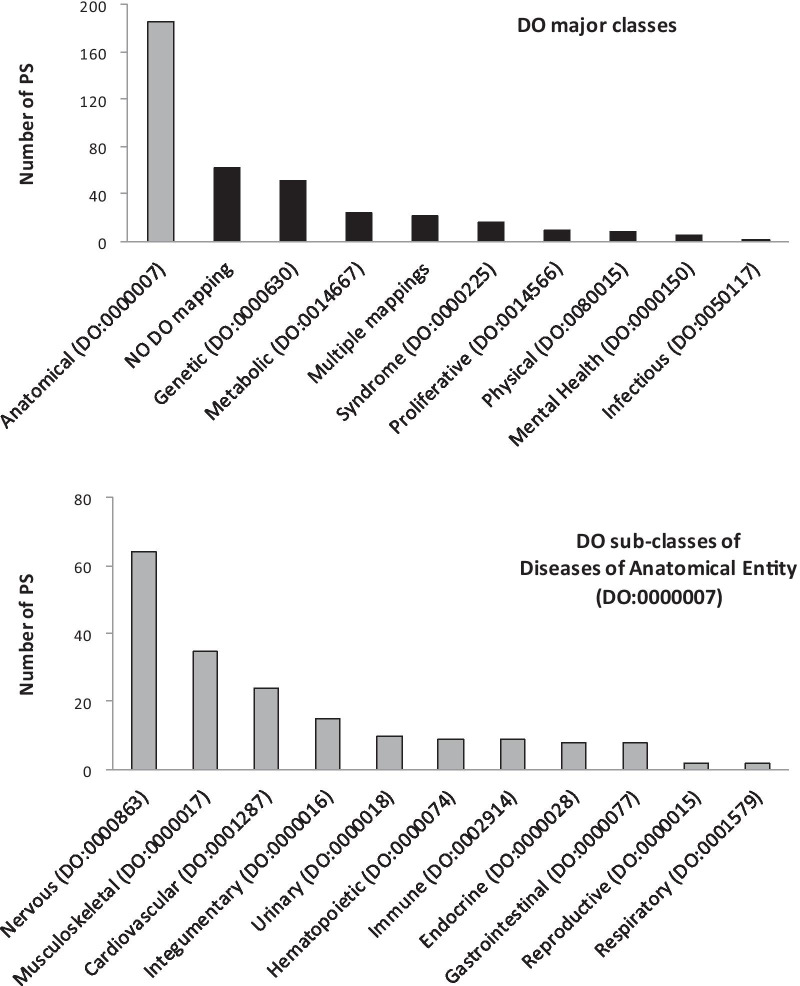
Clinical classification of the PS. Distribution of the PS among the major classes of the DO hierarchy (*top*) and the direct descendants of the class *Disease of anatomical entity* (*bottom*). Note that 62 PS contain D, which have no DO annotations, while 21 PS have more than one DO annotation

Thus, to identify groups of clinically similar D, we retrieved the PS from OMIM. Each PS comprises D, which have been defined similar, in spite of being caused by mutations in different genes. From the full OMIM list of the PS, we focused on a subset of 386 PS and 2,332 D, because these are the D with a known molecular basis. In this subset, the average number of D per PS is 6.2 ± 8.8 (mean ± SD). While 160 PS comprise just 1 or 2 D, the PS *Retinitis pigmentosa* comprises 60 D. Between these extremes, 196 PS comprise from 3 to 15 D, while 29 PS comprise from 16 to 57 D. As a further characterization, we labeled each PS according to the clinical classification of its D according to the DO database. We found that 324 PS contain at least one D annotated with DO terms. Like other ontologies, the DO terms are organized in a hierarchy of increasing coverage and decreasing specificity. Thus, to subdivide the PS into clinical families of reasonably broad coverage (but sufficiently useful specificity), we classified the PS according to the major branches of the DO hierarchy (Fig. 2, *top*). In addition, 186 PS that contain D labeled as *Disease of anatomical entity* could be further subdivided into more specific DO terms that designate specific anatomical systems. The nervous, musculoskeletal and cardiovascular systems were the most represented (*bottom*). Thus, these input data enabled us to focus on thousands of inherited D with known DGP (from the morbid map) and to make correlations with other sources of information, such as their presumptive clinical similarity (PS membership) and clinical classification (DO annotations).

### The HPO database annotates the OMIM-derived D with clinical features (the DP)

As stated above, our major aim was to implement an algorithm that quantifies the within-PS similarity independently on subjective judgment. To this aim, for each pair of D (from either the same or different PS), we quantified their similarity based on the shared DP. To avoid any subjective bias, we retrieved the DP from an independent source, i.e., HPO, which annotates each D with a set of DP. Being an ontology, also HPO arranges its DP terms hierarchically, starting from the root *All*. For simplicity’s sake, however, we regarded as root the more specific term *Phenotypic abnormality* (HP:0000118).

We first retrieved 5,815 DP that annotate the 4,193 D from the morbid map of OMIM. Then, to each DP we attributed a normalized IC, which is inversely proportional to the number of D that the DP annotates in the HPO dataset. For instance, the rather generic (and very frequent) DP *Abnormality of the nervous system* has IC 0.068, whereas the more specific (and less frequent) DP *Hyporeflexia of upper limbs* has IC 1.000. Next, we combined into *4*-tuples all the four inputs, i.e., the D of interest, the PS that the D belongs to, the DP that the D is annotated with and the IC of the DP. We assembled 26,359 *4*-tuples, which encompass 2,118 D, 371 PS and 3,700 DP. On average, each D is annotated with 12.4 ± 11.5 DP, even though there is wide variability, ranging from several D, which are annotated with only 1 DP, to few D, which are annotated with several DP (not shown). Interestingly, 91 PS, which contain the string *syndrome* in their label, are annotated with 21.8 ± 15.3 DP per D, whereas the remaining 280 (non-syndromic) PS have 11.1 ± 8.4 DP per D. Thus, by combining these data into the D-PS-DP-IC *4*-tuples, we assembled the inputs required for quantifying clinical similarity.

### The similarity score quantifies intra-PS similarity based on number and specificity of the shared DP

Thus, we next applied an algorithm that outputs a score of D similarity based on the number and IC of the shared DP. Briefly, for each possible pair of D (D_i_ − D_j_), the algorithm assembles all the pairs of DP that annotate D_i_ and D_j_, in order to identify the shared DP. There are two possible outcomes for each DP pairing, depending on whether the two DP are either identical or different. If identical, the shared DP is the DP itself. If different, the shared DP is the highest specificity ancestor of both DP. Given the hierarchical structure of HPO, even two highly dissimilar DP share, as common ancestor, some low-specificity DP (e.g., the root). Finally, for each D_i_ − D_j_ pair, the algorithm averages the IC of all the shared DP and outputs the similarity score of the two D.

We found that the D_i_ − D_j_ pairs that belong to the same PS have a 4.2-fold higher similarity score than randomly paired D. Thus, an average D_i_, which belongs to a PS composed of *n* D, has an intra-PS similarity score of 0.399 ± 0.107, which is the mean of the pairwise similarities of D_i_ with the remaining *n-1* D in its own PS. In comparison, D_i_ has a random similarity score of 0.094 ± 0.042, which is the mean of the pairwise similarities of D_i_ with *n* − 1 randomly chosen D. Notably, only in 8 (out of 2118) D, the intra-PS similarity score is lower than the random similarity score (not shown). Analysis of the similarity scores by DO class is reported in Fig. S1 (Additional file 1). Thus, by quantifying the intra-PS similarity, we could confirm that the D, which had been placed in the same PS by a subjective judgment of similarity, are similar by objective criteria as well.

### The similarity score identifies the widespread occurrence of inter-PS similarity

The finding that a D is similar to the other D in its own PS does not exclude the possibility that it can be similar to D in other PS as well. Addressing this issue may answer key questions about the global distribution of clinical similarity. Specifically, does the measurement of similarity favor a model of the PS as isolated clinical entities, whose member D are not only similar among themselves but also dissimilar from the D of other PS? Alternatively, does the measurement support a more interconnected model, with numerous similarities among D, even across the borders of the individual PS?

To this aim, we examined all the possible D_i_ − D_j_ pairs (both intra- and inter-PS) and then, for each D_i_, we identified the D_j_ that has the highest similarity score with D_i_. For ease of analysis, we excluded both the D that belong to more than one PS and the PS that contain only one D. As expected, the majority of D (1281, out of 2029, D_i_; 63.1%) have the highest similarity score with a D_j_ in their own PS. Yet, a significant fraction (748 D_i_; 36.9%) have the highest similarity score with a D_j_ in a different PS. For instance, *Adenomas, multiple colorectal* (in PS 175100) has its highest score with *Colorectal cancer, hereditary nonpolyposis, type 2* (in PS 120435). As the example suggests, high inter-PS similarity may occur when two PS, albeit different, share key pathogenic and anatomical features (in this example, altered cell proliferation in the lower intestinal tract).

**Fig. 3:**
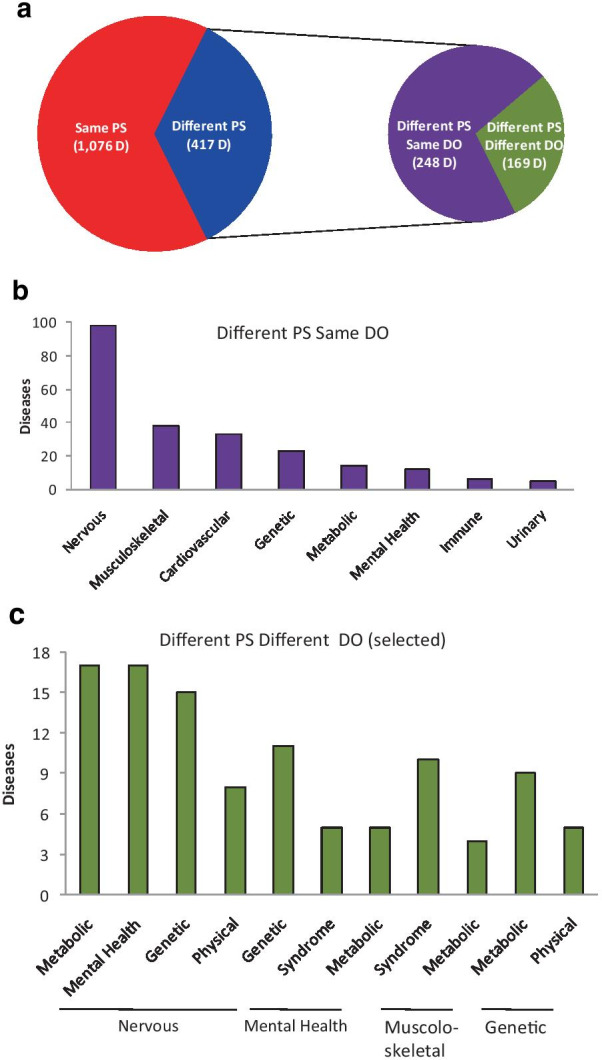
Analysis of inter-PS similarity. **a** Quantification of the D_i_ that have their highest similarity score with another D_j_ in either the same PS (*red*) or a different PS (*blue*) and, in the latter case, quantification of the pairs in which the two D belong to either the same DO class (*purple*) or different DO classes (*green*). **b** Distribution of the DO classes for the D pairs that belong to different PS but to the same DO class. **c** Most common combinations of DO classes for the D pairs that belong to different PS and different DO classes

To investigate the issue further, we examined the pathogenic/anatomical annotations of DO. After excluding the D that either lack or have multiple DO annotations, we confirmed that the majority of D (1076, out of 1493, D_i_; 72.1%; Fig. 3a *red*) have their highest similarity with a D_j_ in their own PS and that a substantial fraction (417 D_i_; 27.9%; *blue*) have their highest similarity with a D_j_ in a different PS. In addition, 248 D_i_ (out of the 417) belong to the same DO class as the D_j_ (*purple*), the most frequent DO class being the nervous system (Fig. 3b). Notably, higher-specificity DO sub-classes were identified within the nervous system class, including 14 retinal D, which belong to different PS (*Leber congenital amaurosis*, *Retinitis pigmentosa* and *Night blindness, congenital stationary*) and share high specificity DP, such as *Nyctalopia*. Remarkably, however, out of the 417 D_i_, the remaining 169 D_i_ belong not only to a different PS than D_j_ but also to a different DO class (Fig. 3a *green*). Interestingly, some heterologous DO combinations were detected frequently (Fig. 3c), the most common involving nervous and metabolic D, possibly reflecting the susceptibility of the developing nervous system to early metabolic defects (Fig. S2; Additional file 1). Thus, by showing that more than one third of D have their highest similarity with a D in a PS other than their own, these data favor a model, in which the PS (in addition to having high intra-PS similarity) also emerge as inter-linked (rather than isolated) clusters of clinical similarity.

### Choosing a network-based representation of the D-DP annotations and D-D similarities

The sheer number of data poses a major challenge to the large-scale display and analysis of the clinical similarities, thereby necessitating a global approach, like the use of networks. As mentioned above, our general procedure consists of assembling the D-DP bipartite graph and then deriving from it the DDSN-C. Nonetheless, the D-DP graph can be assembled by means of different strategies. On one side, one could simply link each D with all the DP that directly annotate the D in HPO. This is, however, a low-sensitivity approach, because it fails to identify as similar those D pairs, whose DP are just subtly different (because the DP occupy contiguous levels in the HPO hierarchy). As a result, the two D will be disconnected in the DDSN-C. On the other side, one could link each D not only with the DP that directly annotate it in HPO, but also with all the ancestors of each annotating DP (down to the root of HPO). This is, however, a low-specificity approach, because it may identify as similar even markedly different D, which share just some poorly informative DP. As a result, the DDSN-C will be a fully inter-connected network of rather limited usefulness.

Thus, we decided to implement an intermediate strategy, which is able to identify occurrences of high clinical similarity within D pairs, even when the two D are not annotated with an identical DP. Specifically, (for any D_i_ − D_j_ pair) we identified—as shared DP_(i,j)_—not only an identical DP that annotates both D_i_ and D_j_ in HPO (*rule 1*) but also a highly similar DP. By ‘highly similar DP’, we refer to two unambiguous criteria. First, DP_(i,j)_ is a first-degree ancestor of the two different DP that annotate D_i_ and D_j_ (*rule 2*). Second, DP_(i,j)_ is the DP that annotates directly D_i_ and is also the first-degree ancestor of the DP that annotates D_j_, or the other way around (*rule 3*). With these three criteria, we identified 1,853,106 distinct *7*-tuples (i.e., D_i_, which belongs to PS_i_ and is annotated with DP_i_, shares DP_(i,j)_ with D_j_, which belongs to PS_j_ and is annotated with DP_j_). There are 624,610 D_i_ − D_j_ pairs, in which 2116 unique D share 3107 unique DP_(i,j)_ (38.1, 42.3 and 19.6% according to rule 1, 2 and 3, respectively). Interestingly, both number and IC of the shared DP_(i,j)_ per D pair are higher, on average, among the pairs, in which D_i_ and D_j_ belong to the same PS, than among the pairs in which D_i_ and D_j_ belong to different PS (Table 1). This way, we assembled the bipartite D-DP graph, with an acceptable compromise of sensitivity and specificity.

**Table 1:** Number and specificity of the DP shared by pairs of HPO-annotated D

Condition	D_i_D_j_ pairs	DP_(i,j)_ count per D_i_D_j_ pair		IC per D_i_D_j_ pair	
		Mean (SD)	Median (range)	Mean (SD)	Median (range)
PS_i_ = PS_j_	15,372	4.8 (4.1)	4 (1–65)	0.38 (0.12)	0.37 (0.08–1)
PS_i_ ≠ PS_j_	609,238	2.5 (2.3)	2 (1–45)	0.28 (0.11)	0.26 (0.07–1)
All	624,610	2.6 (2.4)	2 (1–65)	0.28 (0.11)	0.26 (0.07–1)

The table reports the count and the IC of the DP (as HPO terms), which are shared among the D pairs that are used to assemble the DDSN-C, in the indicated conditions, i.e., when D_i_ and D_j_ are in the same PS (PS_i_ = PS_j_), in different PS (PS_i_ ≠ PS_j_) or both (All)

### The D-DP bipartite graph identifies topologically important D and DP

Besides being prerequisite to the assembly of the DDSN-C, the bipartite D-DP graph provides the opportunity to identify topologically important nodes. Being bipartite, the D-DP graph has two subsets of nodes (D and DP) and one type of edges (D-DP). Thus, the number of nodes (5223) is the sum of the D (2116) and DP (3107) nodes, whereas the number of edges (51,657) is the number of D-DP links. The average connectivity <*k*_*D*_> of the D (i.e., the average number of DP per D) is 24.4 ± 22.5. However, while 138 D are linked to just 1 or 2 DP, a handful of D are linked to hundreds of DP. Examples of highly annotated D include (among others) *Rubinstein-Taybi syndrome 1* (219 DP) and *Cornelia de Lange syndrome 1* (167 DP).

The average connectivity <*k*_*DP*_> of the DP (i.e., the average number of D per DP) is 16.6 ± 34.2. Here, there is high variability as well, because many DP annotate just few D (for instance, 1490 DP annotate less than 6 D), while few DP ‘hubs’ annotate hundreds of D. The DP hubs are rather generic HPO terms like *Neuro-developmental delay* (496 D) and *Intellectual disability* (450 D). A list of the hubs, grouped by the major branches of the HPO tree, is reported in Table S1 (Additional file 2). Notably, the distribution of connectivity *P*(*k*) approximates a power-law, for both *k*_*D*_ and *k*_*DP*_ (Fig. S3; Additional file 1). Having characterized the D-DP graph, we assembled the DDSN-C.

### Deriving the DDSN-C from the bipartite D-DP graph

Deriving the DDSN-C from the D-DP graph requires linking (with an edge) a given pair of D (D_i_ and D_j_), if the two D share one DP. It follows that, if the two D share more than one DP, they will be linked by more than one edge. Actually, the 2116 D of the D-DP graph form 624,610 D_i_ − D_j_ pairs that are linked by 1,626,172 edges in the DDSN-C, with an average of 2.6 ± 2.4 edges per pair. The multiplicity of edges calls for a more compact version of the DDSN-C, to facilitate its visualization and analysis. To this aim, multiple edges linking a pair of nodes were merged into one edge. Furthermore, to preserve the quantitative information on the D_i_ − D_j_ similarity, a weight *w* was assigned to each resulting edge (*w* being the average IC of all the DP that link the two D). The ‘compact’ DDSN-C has still 2116 D nodes but just 624,610 D-D edges (the number of linked D_i_ − D_j_ pairs).

An additional advantage of the compact DDSN-C is the possibility of removing similarities of lower specificity by raising the threshold w* for *w*. Although raising w* reduces both edges and nodes, it is possible to search a w*, at which the maximum number of edges is lost at the expense of a minimal loss of nodes. For instance, applying a w* > 0.45 eliminates 93.2% of the edges but only 6.1% of the nodes (Fig. S4; Additional file 1). Setting a high w* (for instance, w* > 0.80) has also practical usefulness, because it allows to display subgraphs of the DDSN-C (Fig. S5; Additional file 1)—and of the D-DP (Fig. S6; Additional file 1) as well—which (with numerous nodes and edges) would be otherwise difficult to visualize in their entirety. Thus, in conclusion, the complexity of all the D_i_ − D_j_ similarities can be suitably displayed by the compact and weighted version of the DDSN-C.

### Assortative analysis of the DDSN-C

As many D that belong to the same PS have a high degree of similarity, the corresponding nodes in the DDSN-C remain linked to each other even at high w*. Thus, if we regard the PS annotation of each D as a *property* that the corresponding node either possesses or does not possess, then the DDSN-C is *assortative* with respect to that property (the PS). Hereafter, we analyzed how PS assortativity is related to the overall structure of the DDSN-C. The analysis requires first counting all the observed *dyads*, i.e., the pairs of nodes in the DDSN-C in which both D link mutually and belong to the same PS. Conversely, the *anti-dyads* are the pairs of nodes, which link mutually but belong to different PS. Next, one quantifies the number of expected dyads and anti-dyads that one should expect, if the property were distributed randomly. Finally, for each PS, the observed-to-expected ratios of dyads and anti-dyads is calculated to define the *dyadicity D* and the *heterophilicity H* of that PS, respectively. Briefly, if *D* > 1, the PS is *dyadic*, and thus similar D link to each other more frequently than expected. Conversely, if *D* < 1, the PS is *anti-dyadic*. Furthermore, if *H* > 1, the PS is *heterophilic*, and thus dissimilar D link to each other more frequently than expected. Conversely, if *H* < 1, the PS is *heterophobic*.

**Fig. 4:**
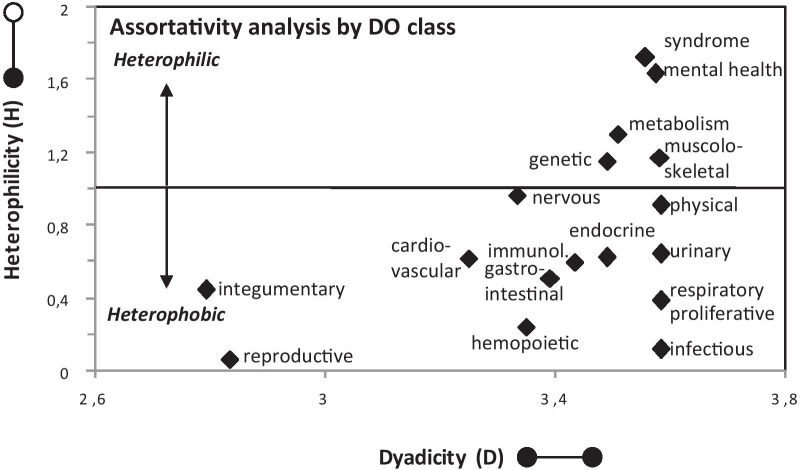
Assortativity analysis of the DDSN-C. The dyadicity to heterophilicity (D/H) scatterplot summarizes the findings of the dyad analysis carried out for the PS-associated D in the DDSN-C. For ease of analysis, the PS have been clustered according to their DO class

Focusing on the 316 PS that contain at least two D, we found that almost all the PS are dyadic (with comparable levels of dyadicity), while only 7 PS (e.g., *Familial episodic pain syndrome*) are anti-dyadic. Remarkably, however, the dyadic PS vary widely in their degree of heterophilicity. On one extreme, we observed highly heterophobic PS, most of which are restricted to a defined anatomical structure (for instance, the tooth, as in *Amelogenesis imperfecta*). On the other extreme, we found highly heterophilic PS, including numerous syndromic PS. Finally, we evaluated whether different DO classes contribute differently to heterophilicity and found that the most heterophilic PS belong to classes that are not well-defined in anatomical terms, including syndromic, mental health and metabolic disorders (Fig. 4). Thus, the assortative analysis indicates that the PS, in addition to being internally similar, also display varying degrees of external similarity, ranging from heterophobic to heterophilic configurations.

### Comparison of the DDSN-C with other networks of D similarity

Finally, we compared the DDSN-C with two other networks of D similarity, the HSDN and the SGPDN [11]. As different vocabularies were used to label the D nodes—OMIM (in DDSN-C) and MeSH (in HSDN and SGPDN)—the OMIM identifiers had first to be converted (whenever possible) into the corresponding MeSH terms. Then, we assembled the DDSN-C*, a ‘deciphering’ subgraph of the DDSN-C whose nodes can be expressed as both OMIM and MeSH terms. The DDSN-C* contains a substantial fraction of the D nodes (86.6%) and D_i_D_j_ edges (69.7%) of the original DDSN-C (Table 2). Finally, we counted how many D pairs, which are linked—because similar—in the DDSN-C, are also linked in the HSDN and in the SGPDN.

**Table 2:** Comparison of the DDSN-C with other networks of D similarity

Networks	Shared	Terms	Nodes	Edges
*Original*				
HSDN	–	MeSH	4216	7,389,705
SGPDN	–	MeSH	1594	132,802
DDSN-C	–	OMIM	2116	624,610
*Deciphering*				
DDSN-C*	–	OMIM	1832	435,539
		MeSH	1306	322,235
*Intersections*				
DDSN-C* $\cap$ HSDN	N	OMIM	1092	122,410
	N and E	OMIM	1081	105,340
DDSN-C* $\cap$ SGPDN	N	OMIM	1060	117,521
	N and E	OMIM	957	39,348

HSDN, Human Symptoms Disease Network; SGPDN, Shared Symptoms and Genes/PPI Disease Network; MeSH, Medical Subject Headings; E, edges; N, nodes; $\cap$, intersection

First, we calculated the intersection of the DDSN-C* with the HSDN. The DDSN-C* contains 1,092 D nodes (as OMIM terms) that have corresponding MeSH term(s) in the HSDN. We found that 86.1% of all the OMIM_i_ − OMIM_j_ edges in the DDSN-C* (105,340 out of 122,410) have a corresponding MeSH_i_ − MeSH_j_ edge in the HSDN. Almost all (105,324) of these edges (where MeSH_i_ ≠ MeSH_j_) are *inter-PS* links. In addition, 8893 OMIM_i_ − OMIM_j_ pairs—though lacking a corresponding MeSH_i_ − MeSH_j_ pair in the HSDN—have the highest possible degree of similarity, because the two D in the pair are designated by an identical MeSH term. Notably, almost all (8861) of these edges (where MeSH_i_ = MeSH_j_) are *intra-PS* links.

Next, we calculated the intersection of the DDSN-C* with the SGPDN, which is smaller than the HSDN, because it is limited to the D pairs (of the HSDN) that share genes or interacting proteins. The DDSN-C* contains 1060 OMIM nodes that have corresponding MeSH term(s) in the SGPDN. These nodes form 117,521 OMIM_i_ − OMIM_j_ edges of D_i_D_j_ similarity. We found that 33.5% of all the OMIM_i_ − OMIM_j_ edges in the DDSN-C* (39,348 out of 117,521, mostly *inter-PS*) have a corresponding MeSH_i_ − MeSH_j_ edge in the SPGDN. As above, numerous OMIM_i_ − OMIM_j_ pairs (8790; mostly *intra-PS*), which lack a corresponding MeSH_i_ − MeSH_j_ pair in the SGDPN, are designated by an identical MeSH term. Thus, in spite of significant differences in scope and method, the DDSN-C has a substantial degree of overlap with these two other networks of D similarity.

## Discussion

This study was undertaken to evaluate whether the available datasets and algorithms enable an objective and quantifiable appraisal of clinical similarity and, if so, to assess the global distribution of similarity among inherited disorders. The major findings are the following. First, pairs of D that belong to the same PS are more similar to each other compared with randomly paired D, so that the corresponding nodes in the DDSN-C link preferentially to each other and almost all the PS achieve high levels of dyadicity. Second, in spite of the high intra-PS similarity, about one third of the D have their highest similarity score with a D that belongs to a different PS, which accounts for the numerous inter-PS links in the DDSN-C. Third, the PS display different types of mutual relations that influence the global structure of the DDSN-C, ranging from almost isolated heterophobic PS (at the periphery of the network) to highly inter-connected heterophilic PS (in the center of the network). We believe that these findings provide reasonable answers to the starting questions. First, they show how applying the similarity-computing algorithms to the available datasets enables an objective measure of clinical similarity among thousands of inherited disorders. Second, the results cast light on the global distribution of the PS-based similarities, with the intriguing observation of strong intra-PS similarity co-existing with varying degrees of inter-PS similarity.

Our approach has three limitations. The first limitation is quantitative, because the databases we rely on are far from being exhaustive. Nonetheless, the sheer amount of data that are available to date (thousands of D annotated with both PS and DP) is a reassurance that the dataset we have assembled (albeit incomplete) is representative of the whole set of inherited D in humans. The second limitation is qualitative, because the wide variability (in number and specificity) of the DP annotations can influence the precision of the computed similarity. For instance, one could overestimate the similarity of two D that share numerous (albeit poorly specific) DP. However, the risk of insufficient precision is limited, because the similarity score, which averages the IC of the shared DP, at the same time takes into account both number and specificity of the DP. Furthermore, considering the steady improvements in coverage and specificity of OMIM and HPO, the forthcoming releases of these databases will lessen these two types of limitations. Finally, a third limitation is that we studied monogenic disorders only, while neglecting polygenic D, as well as pathogenic mutations that result in multi-factorial disorders or in increased susceptibility to infections. In addition, even with the monogenic D, we did not take into account the effect of different mutations that affect the same locus. Apart from the desire of restricting the analysis to a convenient set of D, our decision to focus on monogenic D derives from the general plan of comparing the clinical similarity of the D (this manuscript) with their biological similarity (accompanying manuscript). In this plan, the similarities cannot be but gene-centered, because the biological similarity is based on the functional annotations of the normal gene products, whereas the clinical similarity is based on the phenotypic consequences of the mutated counterparts (the DGP) of the same gene products.

The present study, however, has also many strengths. First, as already mentioned, our relational queries of independent databases have defined similarity in a way that is independent on subjective judgment. Second, the assembly of all the binary similarities into the DDSN-C provides a global view of clinical similarity. Third, dyad analysis links the local level (of the binary D-D similarity) with the global level (of the DDSN-C). Finally, our work complements, in an original manner, previous studies that have been performed to support the process of differential diagnosis, whereby the physician faces the task of choosing the candidate D that best accounts for the set of DP presented by the patient. Clearly, sound judgment remains prerequisite to reaching a definitive diagnosis and suggesting correct therapy. Yet, differential diagnosis in medical genetics remains challenging, because of the vast amount of genetic D and the even greater amount of DP that characterize each D. In addition, many D lack pathognomonic DP and display nonspecific DP, as well as variable expression and penetrance [12].

Furthermore, our findings are strengthened by the substantial intersection (86.1% of shared edges) of the DDSN-C* with the HSDN [11], in spite of clear differences in the type of input (expert knowledge for the DDSN-C *versus* literature mining for the HSDN). Remarkably, the DDSN-C* also displayed a relatively high intersection (33.5%) with the SGDPN [11], even though the SGDPN only contains a rather limited subset of clinical similarities, i.e., those due to presumptively similar biological mechanisms (as inferred from shared genes or protein interactions). The observation that about one third of the DDSN-C* intersects with the SGDPN suggests that several instances of clinical similarity in the DDSN-C are also instances of biological similarity, a conclusion that we have reported in the accompanying manuscript [13].

Among the previous studies that our work complements, we cannot fail to mention the extremely useful databases we have used here. OMIM, in particular, provides a list of more than four thousands inherited D together with their symptoms and signs [6]. Thus, computer-based searches of semantic similarity among the OMIM-encoded D can facilitate differential diagnosis, by comparing a query (the list of DP presented by the patient) with a reference (the list of DP known to be associated with a given D). However, simple text matching is insufficient, because the terms used to design the DP have varying degrees of specificity. Thus, comparison of the query and reference DP should take into account not only their textual identity but also their likeness of meaning, i.e., their semantic content [14]. Achieving this goal requires a representation of all the DP terms as a directed acyclic graph, which describes the DP and their semantic relationships (mostly in a ‘is a’ format). In this respect, a further important development [15] has been the assembly of the HPO database [7]. Also essential has been the implementation of algorithms that identify (as best matching DP for any pair of DP) their most informative common ancestor [16] and calculate (for any pair of D) a similarity score as the average of all the best matching DP [5, 17]. With text-mining algorithms, other studies have extracted numerous D-DP associations [18, 19], which have been used to assemble D-DP networks, in which clinically similar D formed homogeneous clusters [11, 20, 21]. However, all these studies move from the DP to the D to identify either candidate D genes (the differential diagnosis algorithms) or clusters of similar D (the networks). In contrast, our work can be seen as a reverse engineering of these processes, because it moves from already defined clusters of presumptively similar D (the PS) to quantify their mutual (DP-based) degree of clinical similarity.

## Conclusions

In conclusion, the work presented here has the potential usefulness to improve the clinical process of differential diagnosis. In addition, it sets the stage for the analysis of the biological similarity between D, as well as the correlation between clinical and biological similarities, which are the objects of the accompanying manuscript [13].

## Notes

1. Gamba A, Salmona M, Cantù L, Bazzoni G. The similarity of inherited diseases (II): clinical and biological similarity between the phenotypic series. BMC Med Genomics. 2020 Sep 24;13(1):139.

## Supplementary Information

**Additional file 1: Fig. S1** *Intra-PS similarity.* The figure reports the distribution of the number of PS among the high-level DO classes (*grey bars*) as well as the average coefficient of intra-PS similarity (as mean ± SD; *black squares*). Interestingly, the PS belonging to DO classes that are well characterized in anatomical terms (e.g., disorders of the respiratory, reproductive and cardiovascular system) have intra-PS similarity scores above the mean similarity value of all the cumulative DO classes (*dotted line*). In contrast, the PS that are not anatomically defined (for instance, metabolic, infectious, hematologic, psychiatric and endocrine diseases) have intra-PS score lower than the average score. **Fig. S2** *Inter-PS similarity.* The figure reports a selection of D that belong to the DO-classes DO:0014667 (Disease of metabolism; *left side*) and DO:0000863 (Nervous system disease; *right side*) and that are more similar to a D in another PS (in these examples, a metabolic D has the highest similarity with the indicated neurological D). The figure is derived from the DDSN-C. **Fig. S3** *Distribution of the P(k) in the bipartite D-DP graph.* The distribution of connectivity *P*(*k*) approximates a power-law for the nodes indicating both the D (*k*_*D*_; *top*) and the DP (*k*_*DP*_; *bottom*). **Fig. S4** *Raising the thresholds and network fragmentation.* Raising the threshold (w*) in the DDSN-C (A) and in the DP (B) progressively reduces the fraction of D nodes (*white diamonds*), DP nodes (*gray diamonds*), D−D edges (*black squares*) and D-DP edges (*gray squares*). Results are shown as percentage of the total number of nodes and edges in the whole networks (i.e., at a threshold of zero). The *vertical dashed line* indicates the threshold of 0.45 discussed in the text. The *vertical dotted lines* indicate the threshold of 0.80 applied to display the sub-networks of the DDSN-C (Fig. S5) and of the D-DP (Fig. S6). **Fig. S5** A subnetwork of the DDSN-C. In the DDSN-C, applying a threshold for the average IC of the shared DP (w* > 0.80) causes loss of nodes and edges (as reported in Fig. S4 *panel A*), as well as fragmentation of the DDSN-C. The figure shows the remaining giant connected component, which contains 178 D nodes and 2379 D-D edges. The graph can be best visualized on a computer screen at high magnification. **Fig. S6** A sub-network of the D-DP bipartite graph. In the D-DP bipartite graph, applying a threshold for the IC of the DP (w* > 0.80) causes loss of nodes and edges (as reported in Fig. S4 *panel B*), as well as fragmentation of the D-DP graph. The figure shows the remaining giant connected component, which contains 655 nodes (218 D and 437 DP) and 744 D-DP edges. *White circles* and *gray* *rectangles* are D and DP, respectively.

**Additional file 2: Table S1** The DP hubs in the D-DP bipartite graph.

## Data Availability

All the datasets used in this manuscript are publicly available from the following sources. OMIM (https://www.omim.org/downloads/) for the gene map (genemap2.txt) and the morbid map (morbidmap.txt), which can be accessed upon registration without a license. The full list of the PS is available from OMIM on request; HPO (http://www.obofoundry.org/ontology/hp.html) for the HPO vocabulary and hierarchy (hp.obo) and the list of the DP that annotate the D (phenotype_annotation.tab). The annotation list can be downloaded at http://compbio.charite.de/jenkins/job/hpo.annotations/lastStableBuild/artifact/misc/phenotype_annotation.tab; DO (http://purl.obolibrary.org/obo/doid.obo) for the etiological classification of the D; GitHub (https://github.com/alessio-gamba/Similarity_Intra_PS) for the scripts, the D-DP bipartite graph (d_dp.txt) and the DDSN-C (ddsn_c.txt), as well as the average (sim_mean.txt) and maximal (sim_max.txt) similarities.

## Abbreviations

D: Diseases
DDSN-C: Disease-disease similarity network-clinical
DGP: Disease gene products
DO: Disease ontology
DP: Disease phenotype
HPO: Human phenotype ontology
IC: Information content
MeSH: Medical subject headings
OMIM: Online Mendelian inheritance in man
PS: Phenotypic series
