# Supplementary material for: The similarity of inherited diseases (I): clinical similarity within the phenotypic series
Source: BMC Med Genomics. 2021 Feb 23;14:52. doi: 10.1186/s12920-021-00900-7 (PMC7903653; doi:10.1186/s12920-021-00900-7)
Supplement: Supplementary file 1 — Additional file 1: Fig. S1 Intra-PS similarity. The figure reports the distribution of the number of PS among the high-level DO classes (grey bars) as well as the average coefficient of intra-PS similarity (as mean ± SD; black squares). Interestingly, the PS belonging to DO classes that are well characterized in anatomical terms (e.g., disorders of the respiratory, reproductive and cardiovascular system) have intra-PS similarity scores above the mean similarity value of all the cumulative DO classes (dotted line). In contrast, the PS that are not anatomically defined (for instance, metabolic, infectious, hematologic, psychiatric and endocrine diseases) have intra-PS score lower than the average score. Fig. S2 Inter-PS similarity. The figure reports a selection of D that belong to the DO-classes DO:0014667 (Disease of metabolism; left side) and DO:0000863 (Nervous system disease; right side) and that are more similar to a D in another PS (in these examples, a metabolic D has the highest similarity with the indicated neurological D). The figure is derived from the DDSN-C. Fig. S3 Distribution of the P(k) in the bipartite D-DP graph. The distribution of connectivity P(k) approximates a power-law for the nodes indicating both the D (kD; top) and the DP (kDP; bottom). Fig. S4 Raising the thresholds and network fragmentation. Raising the threshold (w*) in the DDSN-C (A) and in the DP (B) progressively reduces the fraction of D nodes (white diamonds), DP nodes (gray diamonds), D−D edges (black squares) and D-DP edges (gray squares). Results are shown as percentage of the total number of nodes and edges in the whole networks (i.e., at a threshold of zero). The vertical dashed line indicates the threshold of 0.45 discussed in the text. The vertical dotted lines indicate the threshold of 0.80 applied to display the sub-networks of the DDSN-C (Fig. S5) and of the D-DP (Fig. S6). Fig. S5 A subnetwork of the DDSN-C. In the DDSN-C, applying a threshold for the average IC of the s [file 12920_2021_900_MOESM1_ESM.pptx]

## Slide 1
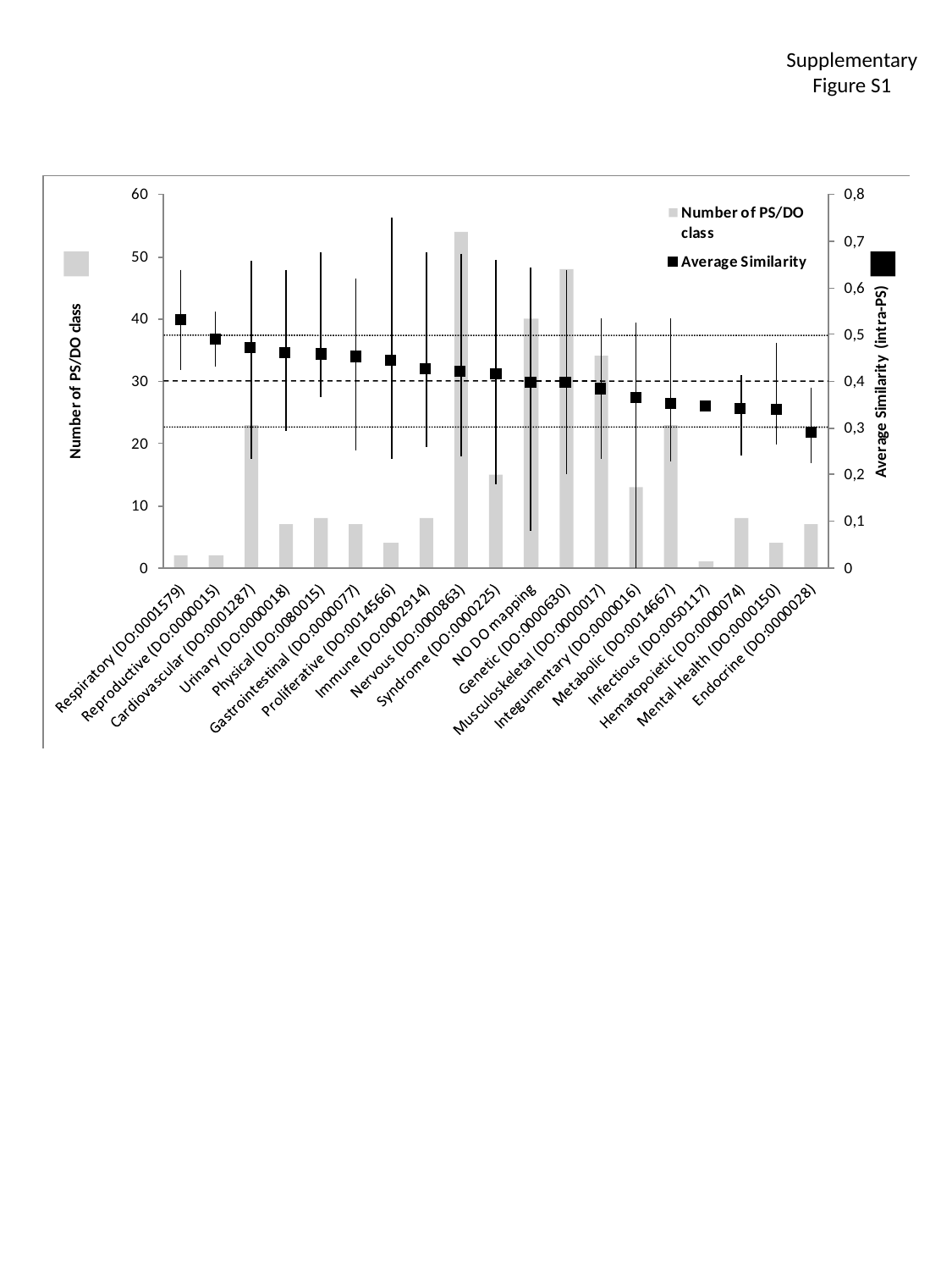

Supplementary
Figure S1

## Slide 2
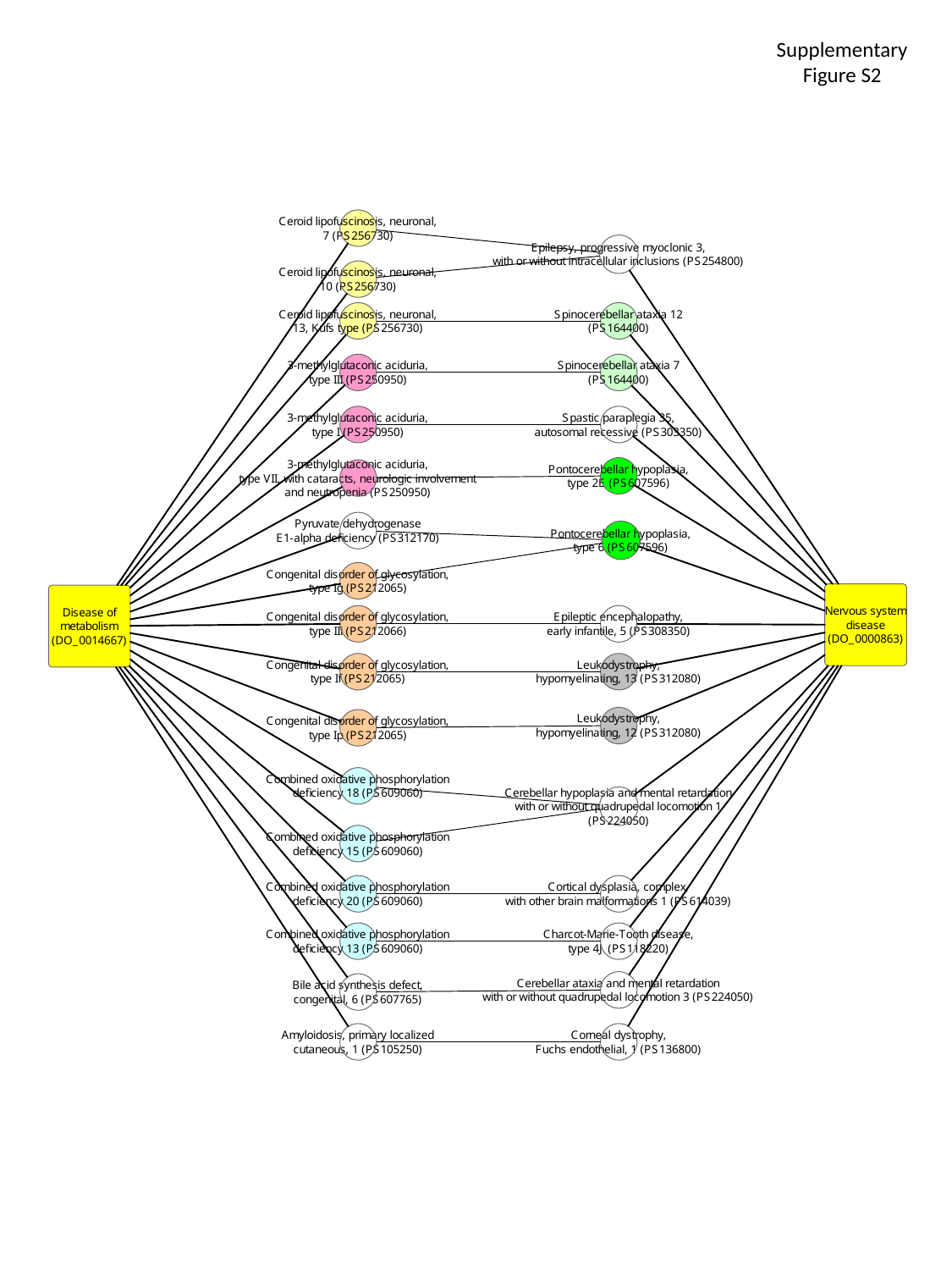

Supplementary
Figure S2

## Slide 3
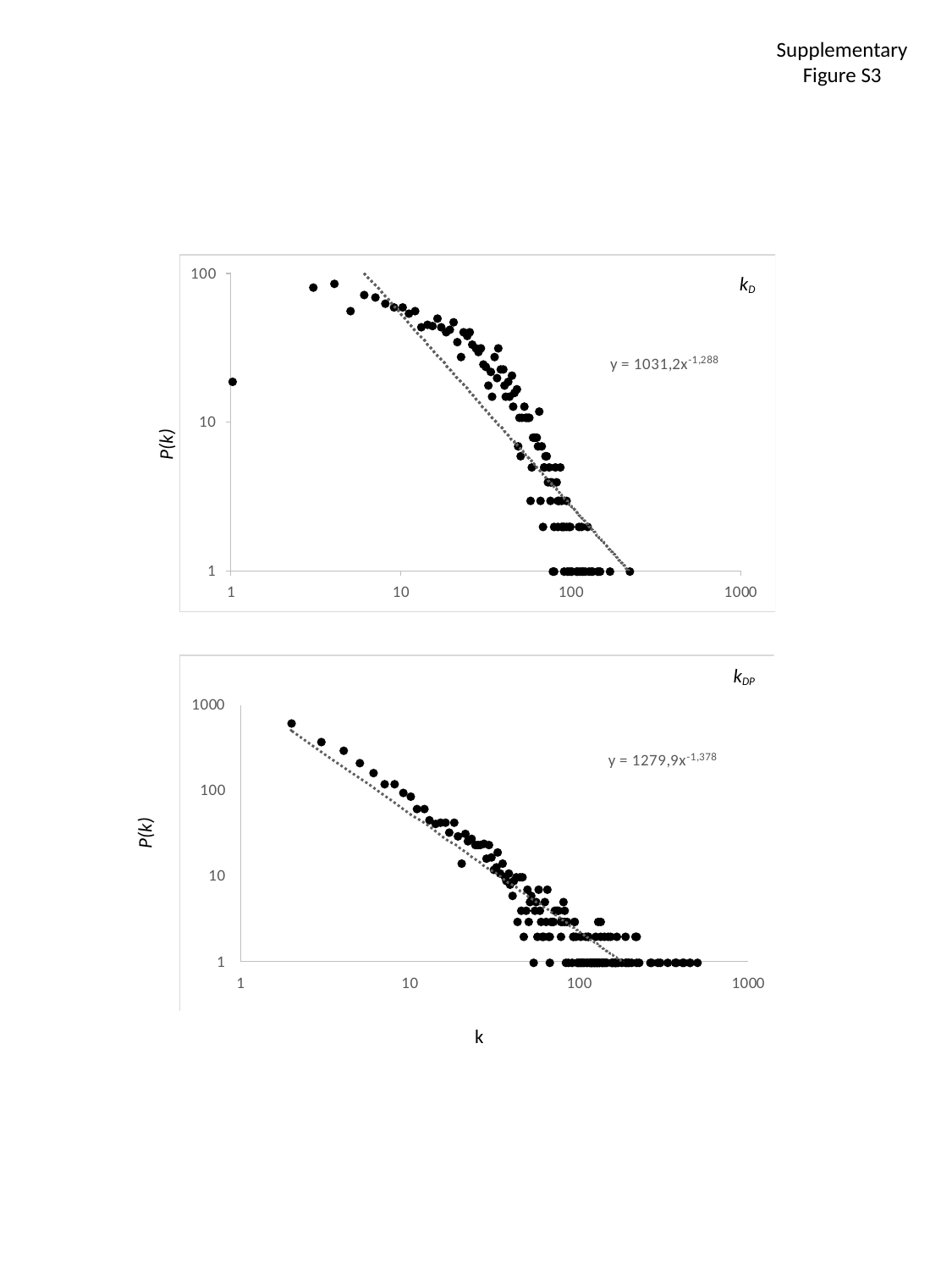

Supplementary
Figure S3
kD
P(k)
kDP
P(k)
k

## Slide 4
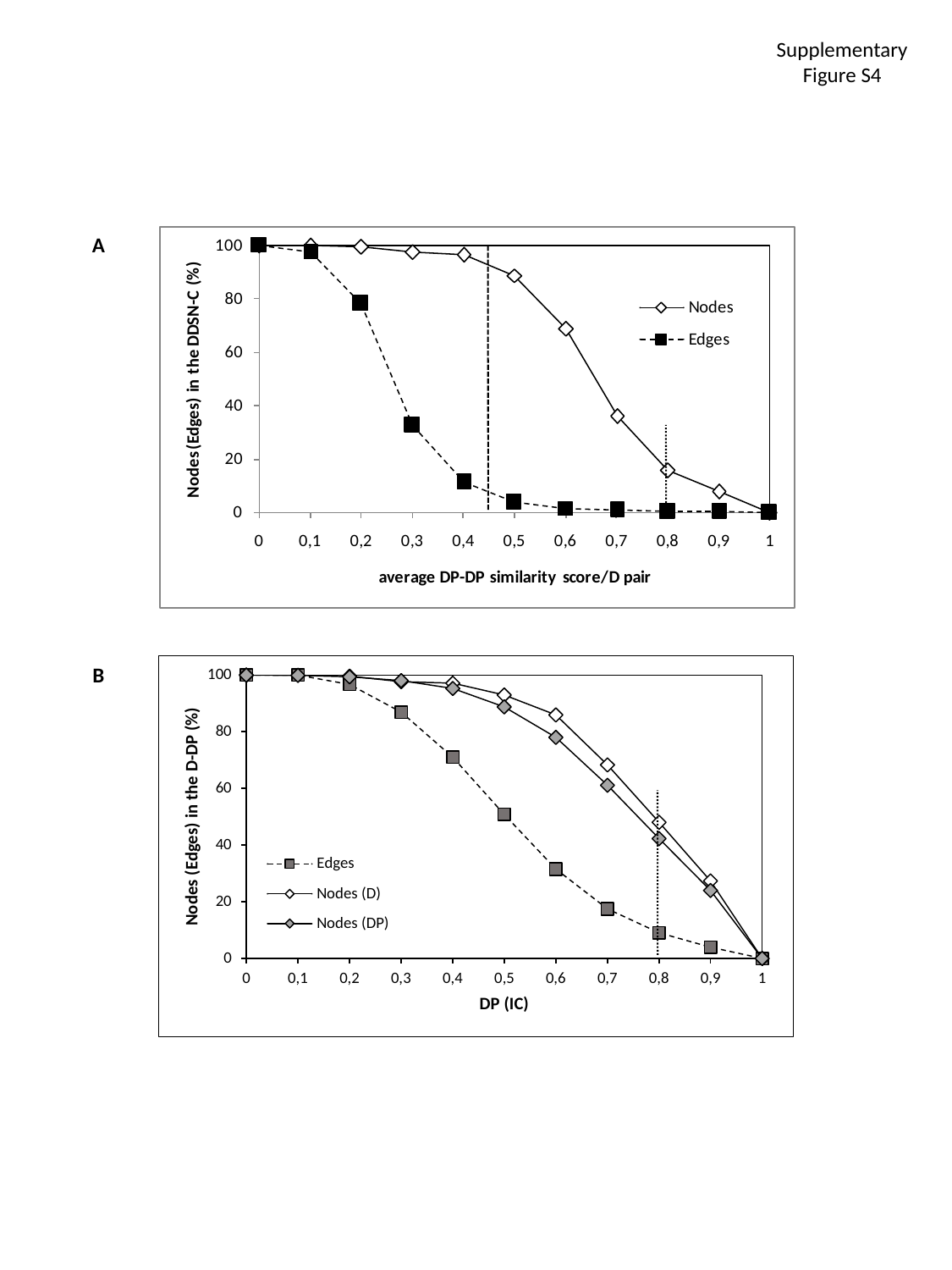

Supplementary
Figure S4
A
B

## Slide 5
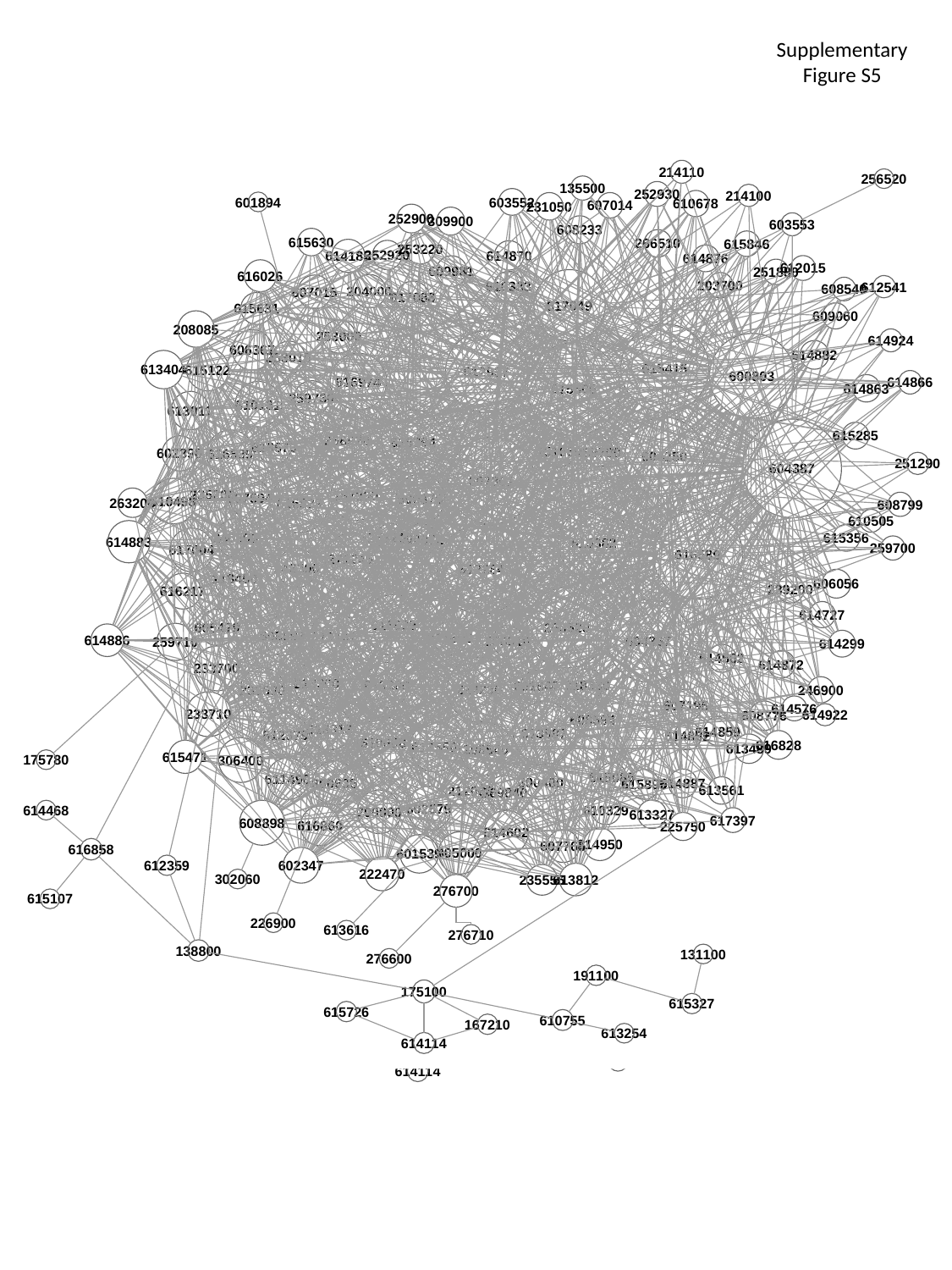

Supplementary
Figure S5

## Slide 6
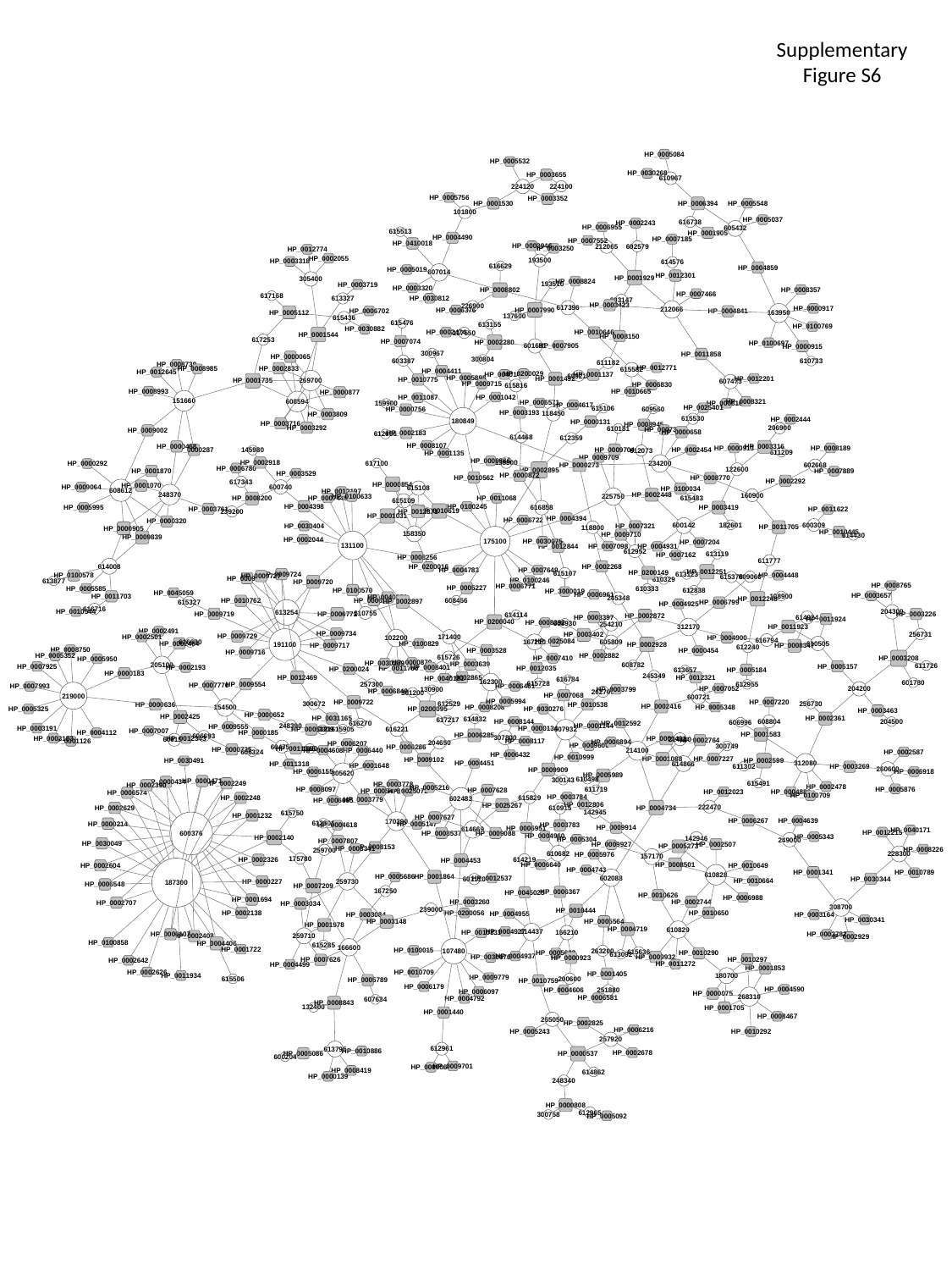

Supplementary
Figure S6
