# Supplementary material for: The similarity of inherited diseases (I): clinical similarity within the phenotypic series
Source: BMC Med Genomics. 2021 Feb 23;14:52. doi: 10.1186/s12920-021-00900-7 (PMC7903653; doi:10.1186/s12920-021-00900-7)
Supplement: Supplementary file 2 — Additional file 2: Table S1 The DP hubs in the D-DP bipartite graph. [file 12920_2021_900_MOESM2_ESM.docx]

**Table S1** *The DP hubs in the D-DP bipartite graph*

| **HPO general class** | **DP (HPO:id)** | **Inter-PS links** | **Intra-PS links** | **D/DP** | **Root** | **IC** |
| --- | --- | --- | --- | --- | --- | --- |
| **Abnormality of the nervous system (HP:0000707)** | Intellectual disability (HP:0001249) | 97,480 | 2,476 | 450 | 5 | 0.187 |
|  | Global developmental delay (HP:0001263) | 80,075 | 2,018 | 406 | 6 | 0.202 |
|  | Seizures (HP:0001250) | 67,092 | 1,748 | 372 | 4 | 0.194 |
|  | Neurodevelopmental delay (HP:0012758) | 63,394 | 962 | 496 | 5 | 0.171 |
| **Abnormality of the musculature (HP:0003011)** | Muscular hypotonia (HP:0001252) | 51,422 | 1,286 | 410 | 5 | 0.196 |
|  | Generalized hypotonia (HP:0001290) | 40,441 | 1,175 | 289 | 6 | 0.233 |
|  | Muscle weakness (HP:0001324) | 20,912 | 844 | 215 | 4 | 0.254 |
|  | Spasticity (HP:0001257) | 15,977 | 843 | 186 | 6 | 0.285 |
| **Abnormality of head or neck (HP:0000152)** | Microcephaly (HP:0000252) | 42,603 | 969 | 297 | 7 | 0.249 |
|  | Abnormality of the forehead (HP:0000290) | 12,255 | 174 | 169 | 5 | 0.277 |
|  | Abnormality of globe location (HP:0100886) | 10,730 | 144 | 187 | 5 | 0.268 |
|  | Abnormality of the nasal bridge (HP:0000422) | 8,779 | 130 | 160 | 6 | 0.293 |
| **Abnormality of the skeletal system (HP:0000924)** | Microcephaly (HP:0000252) | 42,603 | 969 | 297 | 7 | 0.249 |
|  | Scoliosis (HP:0002650) | 16,423 | 476 | 187 | 7 | 0.273 |
|  | Micrognathia (HP:0000347) | 8,208 | 122 | 130 | 7 | 0.323 |
|  | Abnormality of the curvature of the vertebral column (HP:0010674) | 8,022 | 159 | 167 | 6 | 0.259 |
| **Growth abnormality (HP:0001507)** | Growth delay (HP:0001510) | 39,498 | 407 | 333 | 3 | 0.188 |
|  | Short stature (HP:0004322) | 23,075 | 352 | 217 | 4 | 0.23 |
|  | Failure to thrive (HP:0001508) | 11,225 | 236 | 152 | 5 | 0.285 |
|  | Decreased body weight (HP:0004325) | 9,002 | 92 | 192 | 4 | 0.258 |
| **Abnormality of the ear (HP:0000598)** | Hearing impairment (HP:0000365) | 25,707 | 730 | 261 | 5 | 0.229 |
|  | Sensorineural hearing impairment (HP:0000407) | 13,009 | 772 | 167 | 6 | 0.289 |
|  | Low-set ears (HP:0000369) | 6,189 | 133 | 113 | 6 | 0.331 |
|  | Abnormality of the pinna (HP:0000377) | 4,635 | 93 | 107 | 5 | 0.317 |
| **Abnormality of the eye (HP:0000478)** | Nystagmus (HP:0000639) | 24,310 | 528 | 224 | 6 | 0.281 |
|  | Abnormality of vision (HP:0000504) | 13,290 | 996 | 178 | 4 | 0.256 |
|  | Abnormality of globe location (HP:0100886) | 10,730 | 144 | 187 | 5 | 0.268 |
|  | Visual impairment (HP:0000505) | 9,318 | 309 | 143 | 5 | 0.322 |
| **Abnormality of the digestive system (HP:0025031)** | Abnormal liver morphology (HP:0410042) | 9,969 | 209 | 156 | 5 | 1 |
|  | Abdominal symptom (HP:0011458) | 7,862 | 103 | 152 | 4 | 0.244 |
|  | Feeding difficulties (HP:0011968) | 7,377 | 273 | 133 | 5 | 0.315 |
|  | Visceromegaly (HP:0003271) | 6,515 | 145 | 127 | 4 | 0.31 |
| **Abnormality of limbs (HP:0040064)** | Abnormality of the foot (HP:0001760) | 7,977 | 302 | 163 | 4 | 0.213 |
|  | Pes cavus (HP:0001761) | 4,409 | 1,051 | 105 | 5 | 0.404 |
|  | Talipes equinovarus (HP:0001762) | 2,696 | 78 | 75 | 7 | 0.378 |
|  | Abnormality of digit (HP:0011297) | 2,436 | 57 | 79 | 5 | 0.213 |
| **Abnormality of the cardiovascular system (HP:0001626)** | Cardiomyopathy (HP:0001638) | 6,905 | 161 | 133 | 6 | 0.33 |
|  | Atrial septal defect (HP:0001631) | 2,658 | 55 | 75 | 6 | 0.398 |
|  | Ventricular septal defect (HP:0001629) | 2,657 | 44 | 74 | 7 | 0.389 |
|  | Arrhythmia (HP:0011675) | 2,127 | 65 | 69 | 5 | 0.36 |
| **Abnormality of the immune system (HP:0002715)** | Recurrent infections (HP:0002719) | 6,849 | 514 | 128 | 4 | 0.3 |
|  | Splenomegaly (HP:0001744) | 2,271 | 75 | 69 | 5 | 0.378 |
|  | Recurrent respiratory infections (HP:0002205) | 2,016 | 216 | 68 | 5 | 0.369 |
|  | Increased inflammatory response (HP:0012649) | 1,449 | 62 | 58 | 5 | 0.291 |

**Table S1** *(continued)*

| **HPO general class** | **DP (HPO:id)** | **Inter-PS links** | **Intra-PS links** | **D/DP** | **Root** | **IC** |
| --- | --- | --- | --- | --- | --- | --- |
| **Abnormality of the genitourinary system (HP:0000119)** | Cryptorchidism (HP:0000028) | 5,613 | 165 | 108 | 8 | 0.33 |
|  | Abnormal renal morphology (HP:0012210) | 2,787 | 28 | 82 | 6 | 0.28 |
|  | Renal cyst (HP:0000107) | 1,453 | 86 | 57 | 7 | 0.441 |
|  | Functional abnormality of the bladder (HP:0000009) | 1,270 | 192 | 58 | 6 | 0.432 |
| **Abnormality of the respiratory system (HP:0002086)** | Respiratory insufficiency (HP:0002093) | 5,397 | 167 | 111 | 4 | 0.346 |
|  | Functional respiratory abnormality (HP:0002795) | 3,192 | 23 | 94 | 3 | 0.262 |
|  | Recurrent respiratory infections (HP:0002205) | 2,016 | 216 | 68 | 5 | 0.369 |
|  | Recurrent upper respiratory tract infections (HP:0002788) | 689 | 165 | 44 | 6 | 0.485 |
| **Abnormality of metabolism/homeostasis (HP:0001939)** | Elevated serum creatine phosphokinase (HP:0003236) | 4,882 | 254 | 102 | 6 | 0.382 |
|  | Acidosis (HP:0001941) | 2,384 | 278 | 80 | 4 | 0.334 |
|  | Abnormality of metabolism/homeostasis (HP:0001939) | 1,005 | 6 | 46 | 2 | 0.136 |
|  | Lactic acidosis (HP:0003128) | 866 | 169 | 46 | 5 | 0.405 |
| **Abnormality of connective tissue (HP:0003549)** | Flexion contracture (HP:0001371) | 3,128 | 84 | 81 | 3 | 0.293 |
|  | Hernia of the abdominal wall (HP:0004299) | 1,085 | 44 | 52 | 4 | 0.393 |
|  | Abnormality of connective tissue (HP:0003549) | 991 | 19 | 81 | 2 | 0.228 |
|  | Umbilical hernia (HP:0001537) | 415 | 20 | 30 | 5 | 0.47 |
| **Abnormality of the integument (HP:0001574)** | Abnormal eyebrow morphology (HP:0000534) | 1,719 | 68 | 65 | 5 | 0.366 |
|  | Abnormal hair quantity (HP:0011362) | 1,561 | 18 | 68 | 5 | 0.343 |
|  | Sparse hair (HP:0008070) | 1,156 | 50 | 51 | 6 | 0.405 |
|  | Abnormality of the nail (HP:0001597) | 921 | 40 | 47 | 4 | 0.351 |
| **Abnormality of blood and blood-forming tissues (HP:0001871)** | Thrombocytopenia (HP:0001873) | 1,508 | 86 | 57 | 5 | 0.408 |
|  | Anemia (HP:0001903) | 1,416 | 66 | 55 | 4 | 0.339 |
|  | Decreased antibody level in blood (HP:0004313) | 875 | 132 | 46 | 6 | 0.463 |
|  | Neutropenia (HP:0001875) | 484 | 44 | 33 | 6 | 0.491 |
| **Abnormality of the endocrine system (HP:0000818)** | Hypogonadism (HP:0000135) | 1,194 | 83 | 55 | 4 | 0.42 |
|  | Puberty and gonadal disorders (HP:0008373) | 574 | 21 | 42 | 3 | 0.341 |
|  | Hypothyroidism (HP:0000821) | 315 | 8 | 27 | 5 | 0.494 |
|  | Primary amenorrhea (HP:0000786) | 257 | 68 | 26 | 7 | 0.501 |
| **Abnormality of prenatal development or birth (HP:0001197)** | Abnormality of the amniotic fluid (HP:0001560) | 1,166 | 21 | 63 | 3 | 0.42 |
|  | Polyhydramnios (HP:0001561) | 789 | 31 | 41 | 4 | 0.466 |
|  | Decreased fetal movement (HP:0001558) | 486 | 32 | 33 | 4 | 0.468 |
|  | Abnormality of prenatal development or birth (HP:0001197) | 316 | 6 | 31 | 2 | 0.342 |
| **Constitutional symptom (HP:0025142)** | Pain (HP:0012531) | 1,040 | 6 | 50 | 3 | 0.403 |
|  | Urinary incontinence (HP:0000020) | 292 | 143 | 30 | 5 | 0.536 |
|  | Myalgia (HP:0003326) | 160 | 10 | 19 | 4 | 0.541 |
|  | Impaired continence (HP:0031064) | 53 | 31 | 30 | 4 | 0.536 |
| **Abnormality of the voice (HP:0001608)** | Abnormality of the voice (HP:0001608) | 655 | 3 | 40 | 2 | 0.411 |
|  | Dysphonia (HP:0001618) | 125 | 4 | 18 | 3 | 0.632 |
|  | Nasal speech (HP:0001611) | 53 | 13 | 12 | 3 | 0.561 |
|  | High pitched voice (HP:0001620) | 44 | 1 | 10 | 3 | 0.627 |

**Table S1** *(continued)*

| **HPO general class** | **DP (HPO:id)** | **Inter-PS links** | **Intra-PS links** | **D/DP** | **Root** | **IC** |
| --- | --- | --- | --- | --- | --- | --- |
| **Abnormal test result (HP:0500014)** | Prolonged QT interval (HP:0001657) | 130 | 79 | 21 | 6 | 0.622 |
|  | Abnormal QT interval (HP:0031547) | 95 | - | 24 | 5 | 0.601 |
|  | Shortened QT interval (HP:0012232) | 6 | 4 | 5 | 6 | 0.811 |
|  | Prolonged PR interval (HP:0012248) | 5 | 1 | 4 | 6 | 0.837 |
| **Neoplasm (HP:0002664)** | Neoplasm of the skin (HP:0008069) | 112 | - | 16 | 4 | 0.469 |
|  | Hematological neoplasm (HP:0004377) | 72 | 5 | 14 | 3 | 0.469 |
|  | Leukemia (HP:0001909) | 26 | 10 | 9 | 4 | 0.525 |
|  | Neoplasm of the breast (HP:0100013) | 19 | 11 | 9 | 4 | 0.601 |
| **Abnormality of the breast (HP:0000769)** | Wide intermamillary distance (HP:0006610) | 61 | 5 | 12 | 6 | 0.597 |
|  | Gynecomastia (HP:0000771) | 45 | 21 | 12 | 4 | 0.567 |
|  | Neoplasm of the breast (HP:0100013) | 19 | 11 | 9 | 4 | 0.601 |
|  | Breast carcinoma (HP:0003002) | 17 | 11 | 8 | 5 | 0.605 |
| **Abnormal cellular phenotype (HP:0025354)** | Defective T cell proliferation (HP:0030253) | - | 1 | 2 | 6 | 0.919 |

For each major class of the HPO hierarchy (*first column*), the table reports the most common DP (*second column*) that link the D within the bipartite D-DP graph. The major classes are the direct descendants of the term *Phenotypic abnormality* (HP:00000118), i.e., the terms with a distance of two from the root *All* (HP:0000001) of the ontology. Both the major classes and the DP are expressed as HPO names (and identifiers, in *brackets*). In addition, for each DP term, the table reports the number of inter-PS (*third column*) and intra-PS (*fourth column*) links, as well as the number of annotated D (*fifth column*), the distance from the root (*sixth column*) and the normalized IC (*seventh column*).
